# Age-specific changes in the serum proteome of female anadromous, hilsa *Tenualosa ilisha:* a comparative analysis across developmental stages

**DOI:** 10.3389/fimmu.2024.1448627

**Published:** 2024-10-18

**Authors:** Hena Chakraborty, Hirak Jyoti Chakraborty, Basanta Kumar Das, Joydev Maity

**Affiliations:** ^1^ Center for NMCG (National Mission for Clean Ganga), Indian Council of Agricultural Research (ICAR)-Central Inland Fisheries Research Institute, Barrackpore, West Bengal, India; ^2^ Department of Fisheries Science, Vidyasagar University, Midnapore, West Bengal, India

**Keywords:** hilsa, proteomics, protein, STRING, Liquid Chromatography-tandem Mass Spectrometry, network analysis, Kyoto Encyclopedia of Genes and Genomes

## Abstract

**Introduction:**

The proteome profile of the female *Tenualosa ilisha* (Hamilton, 1822), a species of great ecological and economic importance, across various age groups was investigated to comprehend the functional dynamics of the serum proteome for conservation and aquaculture, as well as sustain the population.

**Methods:**

Advanced liquid chromatography-tandem mass spectrometry LC-MS/MS-based proteomic data were analysed and submitted to the ProteomeXchange Consortium via PRIDE (PRoteomics IDEntifications database). Bioinformatics analysis of serum proteome have been done and it showed different proteins associated with GO Gene Ontology () terms, and the genes associated with enriched KEGG (Kyoto Encyclopedia of Genes and Genomes) pathways (such as phagosome, mTOR, Apelin signalling pathways, herpes simplex virus) implicated in immune responses.

**Results:**

The expression levels of important immunological proteins, such as those involved in cellular defence and inflammatory responses, were significantly different age-dependently. In this study, we annotated 952, 494, 415, and 282 proteins in year classes IV, III, II, and I Hilsa, respectively, and analysed their Protein–Protein Interaction (PPI) networks based on their functional characteristics. From year classes I to IV, new proteins appeared and were more than three-fold. Notably, class I hilsa displayed a lower abundance of proteins than class IV hilsa.

**Discussion:**

This is the first study, to the best of our knowledge, to report the analysis of the serum proteome of hilsa at different developmental stages, and the results can help improve the understanding of the mechanisms underlying the different changes in protein enrichment during migration in hilsa. This analysis also offers crucial insights into the immune system for hilsa conservation and management.

## 1 Introduction

The hilsa fishery is the largest and most profitable fishery in Bangladesh (1) and significant in several other countries, such as India, Myanmar, Malaysia, Vietnam, Iran, Kuwait, and Iraq (2). It is popular in Bengal, Bihar, Odisha, and Assam from the social, cultural, and religious perspectives. According to Hossain et al. (3), 12.5% fish caught in West Bengal, India, are hilsa. However, in India, a the wild population has drastically declined owing to anthropogenic factors such as overexploitation, habitat destruction, pollution, and dam construction (4).

Proteomics is an advanced method for thoroughly cataloguing whole protein complements and a desirable analytical tool for the large-scale identification of protein alterations in health and disease (5). Mass spectrometry-based proteomics examines global protein composition, post-translational modifications, and the dynamic nature of expression levels. Proteomics is an essential tool for formulating hypotheses in modern biology, because it generates enormous protein expression level data (6, 7). Information on proteomes in aquaculture is essential to improve fish growth and study behavioural changes, flesh quality, and biochemical changes (8–10). Proteomic studies have shown that the most prevalent macromolecules in biological systems can be found in many forms, such as structural proteins, enzymes, hormones, antibodies, and receptor proteins. Each protein form has a specific purpose in critical bodily processes, such as critical amino acid provision and muscle growth and maintenance. The Bottom–Up technique (11) is now the most widely used proteomics workflow, in which protein samples are enzymatically digested and LC-MS/MS examines the resultant peptides. Datasets identifying many proteins were produced using the shotgun approach. The resultant protein lists are frequently analysed for studying protein–protein interactions, pathway analysis, and gene ontology (GO) for biological and molecular processes (12, 13). Proteomic information for the commercially important fish hilsa muscle and nutrition-sensitive protein identification were obtained by Mohanty et al. (14). Hilsa availability has significantly declined owing to anthropogenic factors, particularly indiscriminate fishing, pollution, riverine system obstruction by barrages and dams, and climate change. Therefore, the discovery and bioinformatics phases were the two sequential processes combined in this study. In the first step, a reference dataset for the hilsa fish serum proteome was created using a shotgun Bottom–Up proteomic technique (Discovery Phase). Subsequently, the reference proteome was subjected to a series of bioinformatics investigations, including GO, pathway analysis, and network analysis in the second-year class (Bioinformatics Phase).

In this study, the female hilsa serum proteome was analysed, as the serum is an ideal tissue for understanding the underlying significant biomolecular mechanisms. Serum can provide detailed information on the orchestra of proteins that play essential roles in the body functions of an organism. For instance, the average serum protein concentration in this study was 1.26 mg/mL, indicating that standard techniques cancan readily quantify a single drop of blood containing an individual protein of interest. In addition, hilsa covered a vast salinity (ppt: parts per thousands) variation during spawning migration from the sea (≥35 ppt) to coastal and estuarine water (22.4–33.4 ppt) and then fresh water in the river (0.05 ppt). Moreover, starvation is another factor affecting the physiological immune system during migration (15). Starvation reduces the expression of genes associated with immune proteins (16), and sexual maturity and spawning alter humoral immunity through the interaction of sexual hormones with the immune system (17). *Tenualosa ilisha* illustrates a decrease in immune indicators during its migration, which could result from hormonal and environmental impacts (18). Therefore, physiological, biochemical, and nutritional information are required. Over the years, breeding has successfully produced high-value species for aquaculture. However, conserving this species, or bringing it to a marketable size, remains challenging. Several attempts for artificial breeding and domestication have been made, but hilsa has failed owing to a lack of knowledge. Moreover, breeding, producing, and expanding captive brood stocks have not yielded any appreciable results to date (19). Proteomics study will aid in increasing the potential for using information on the significant fish proteins in protein functional profiling and understanding the changes that take place in the various age groups of the fish behavioural changes, information of protein leads to female maturation during the breeding and migrating processes. Therefore, a complete serum proteome functional study is important to understand this significant immunological stress and breeding process of fish, particularly during spawning migration to fresh water, which will be useful for artificial breeding and captivity.

## 2 Materials and methods

### 2.1 Specimen collection

Wild stock hilsa were collected by a specifically designed lift net during the upstream migration at Farakka, Murshidabad, West Bengal from river Ganga (24°48′1.34″N; 87°55′21.53″E). Fish were anaesthetized using 120 mg/L MS-222 as reported by Neiffer and Stamper (20). Body weight was measured, and the fish were divided into different year classes, such as 100–200 g female hilsa denoted as year class I, 201–300 g denoted as year class II, 301–400 g denoted as year class III, and 401–500 g denoted as year class IV, following the protocol of Rahman et al. (21) (**Table 1**). Females were generally larger than males during the intersex period, and could be distinguished by the abdomen shape or dissection and examination. Blood was collected following a standard protocol (22) by puncturing the caudal vasculature with 2 mL sterile saline. The blood samples were transferred to 1.5 mL microcentrifuge tubes and allowed to clot at room temperature for 2–3 h to collect the serum from the supernatant. For higher yields, the remaining coagulated blood was centrifuged for 5–8 min at 8000 rpm and the same procedure was repeated. Then the serum was brought to the laboratory in the ice bucket, and promptly refrigerated at −40 °C. Finally, for each year class, serum form six fish of the same weight group was pooled and studied using the LC-MS/MS analysis.

**Table 1:** LC-MS and UniProt. Summery.

Hilsa Sample	H3	H28	H29	H34
**Weight range (g)**	401–500	301–400	201–300	100–200
**Length range (cm)**	39.8–40.8	33.7–34.9	25.4–27.8	18.9–21.1
**Year class (no.)**	4	3	2	1
**Total proteome (no.)**	8631	3326	2941	2088
**UniProt mapping (no.)**	952	494	415	282

### 2.2 LC-MS/MS analysis

**Figure 1:**
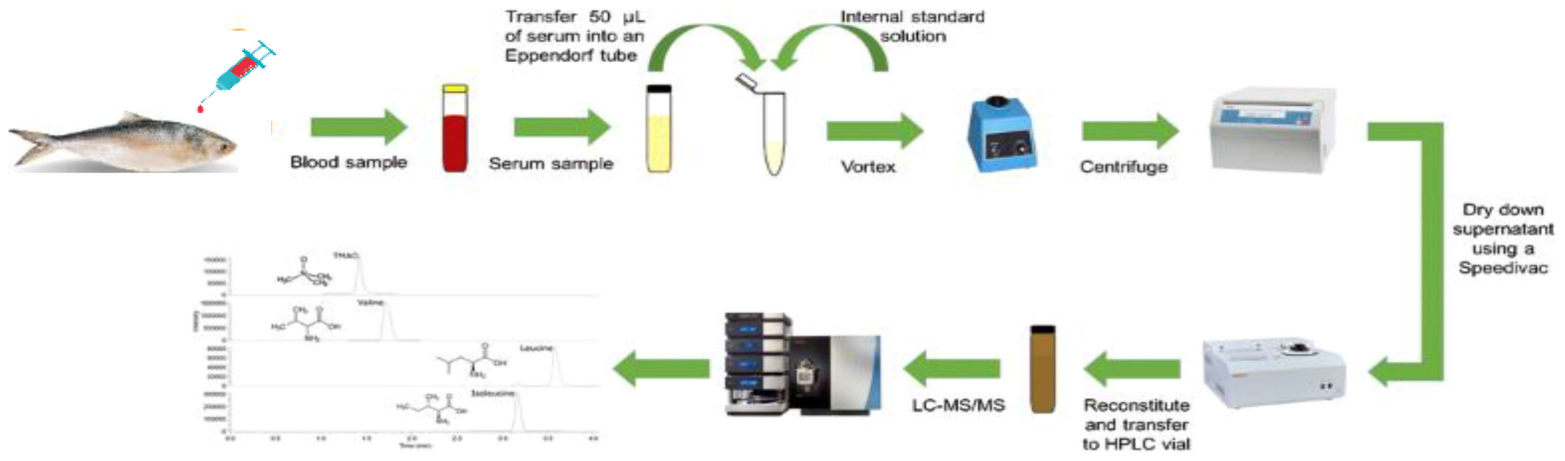
Proteomics of Hilsa peptide sequence process derived by LC-MS/MS.

Liquid chromatography was performed using ACQUITY UPLC equipment from Waters Corporation (USA). The sample (100 µg) was diluted with 100 mM NH_4_HCO_3_ and then treated with 250 mM dithiothreitol at 95 °C for 1 h followed by 250 mM iminodiacetic acid at room temperature in dark for 45 min. Subsequently, the sample was digested in trypsin and separated using an ACQUITY UPLC BEH C18 column (150 mm × 2.1 mm × 1.7 m; Waters). A gradient elution program was used to complete chromatographic separation using mobile phases A and B (0.1% formic acid in water and acetonitrile, respectively). A SYNAPT G2 QTOF instrument (Waters, USA) equipped with an ESI source was used for mass spectrometric detection. The acquisition mass range was set as follows: start mass of 300.000 Da and end mass of 1700.000 Da. Raw mass spectrometry data were processed using the Protein Lynx Global Server (PLGS) 3.0.2, a database search tool (**Figure 1**), and submitted to the ProteomeXchange Consortium via PRIDE (23–25).

### 2.3 Computational proteomics

Using expert-driven algorithms for proteomic data interpretation, high-throughput mass spectrometry-based proteomic data (identifiers) were mapped and annotated using the UniProtKB Fish Proteome Sequence Database (www.uniprot.org). Additionally, network analysis was carried out using the web interface STRING (https://string-db.org/) to understand the protein–protein associations with respect to their enriched functional dynamics. Our in-house hilsa proteome data were mapped against STRING background database of *Danio rerio*. Network topology was analysed using Cystoscope (V-3.10.2). GO annotations such as ‘molecular functions’, ‘cellular components’, and ‘biological processes’, as well as KEGG pathway enrichment analysis, were carried out using the R packages. Venn diagram analysis was performed to segregate unique and common GO terms with respect to different age groups using Venn-2.1.

## 3 Results

### 3.1 LC-MS/MS sequencing of hilsa serum

**Figure 2:**
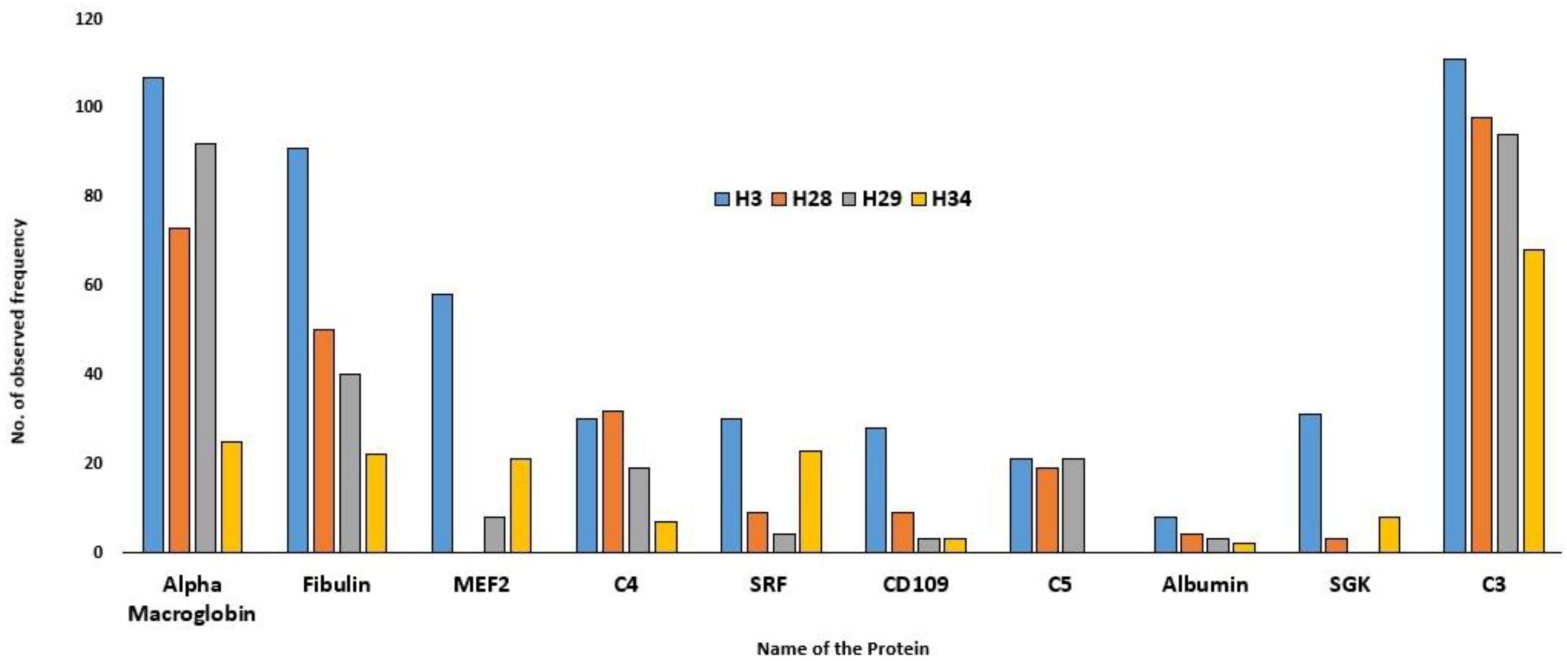
Top Ten observed protein frequency in Hilsa female.

The results of LC-MS are summarised in **Table 1**. The total proteome of female hilsa was identified with respect to its physicochemical properties, such as description, MW (Molecular weight), PLGS (ProteinLynx Global Server), peptides, and coverage (%) (**Supplementary File 3**). A total of 8631 (maximum), 3326, 2941, and 2088 proteomes were quantified from the samples year class IV (H3), year class III (H19), year class II (H28), and year class I (H29) by LS-MS sequencing, respectively, and their PLGS score were 4.08–1274.28, with the maximum coverage of 88.51% in year class IV hilsa. These data were further screened with UniProt mapping, and overall, 952 proteins were revealed in year class IV hilsa, out of which 29 were uncharacterized, followed by 494 (uncharacterized: 20), 415 (uncharacterized: 17), and 282 (uncharacterized: 9) proteins (**Table 1**). Approximately 3–4% identified proteome comprises these unnamed or uncharacterized proteins. The top 10 most observed proteins (**Figure 2**) and their functions in female hilsa are listed in **Supplementary Table 1**, and these were alpha2 macro-globin, fibulin, mef2, complement proteins (C3 C4, C5), albumin, serum response factor, cd109, and non-specific serine/threonine protein kinase. Alpha2 macro-globin, fibulin, and C3 were predominant.

### 3.2 GO enrichment analysis

**Figure 3:**
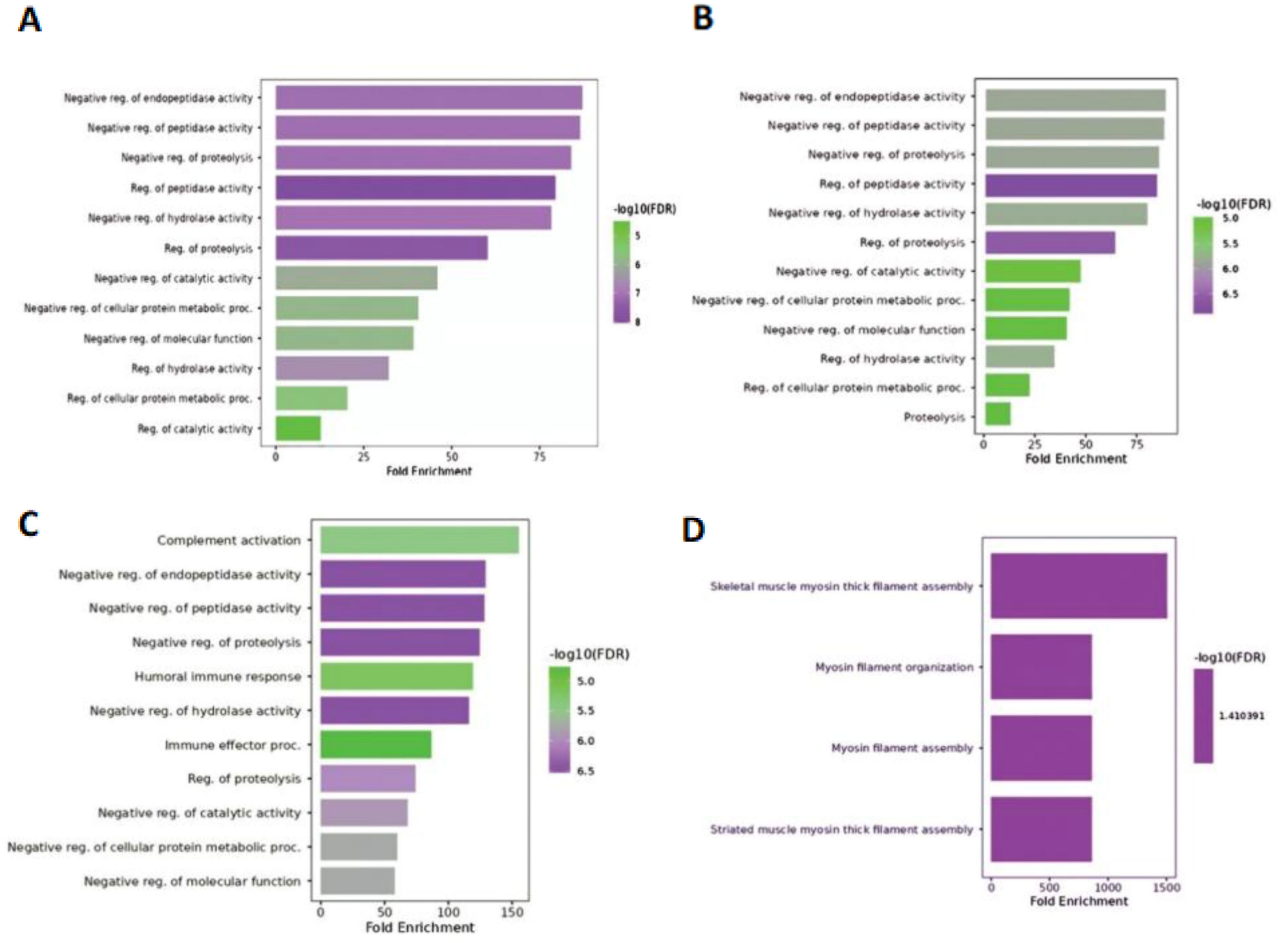
Biological function of 4 samples; **(A)** denotes- sample H3 (IV-year class), **(B)** denotes sample H28 (III-year class), **(C)** denotes sample H29 (II-year class) and **(D)** denotes- sample H34 (I-year class).

GO analysis using three categories (biological processes, molecular function, and cellular components) revealed that in hilsa year class I, four GO terms were enriched (**Figure 3D**) in biological processes such as myosin-related filament assembly and organization (highest fold enrichment value found in skeletal muscle and lowest in striated muscle). In Class II, 11 GO terms were primarily responsible for GO study. The highest enrichment value was observed for complement activation and negative regulation of molecular functions (**Figure 3C**). In addition, when comparing hilsa year classes III and IV, 12 main biological functions were overrepresented with negative regulation of endopeptidase activity and minimum found in proteolysis (**Figure 3B**) and regulation of catalytic activity (**Figure 3A**) with high −log10 false discovery rate (FDR) 5.0, respectively.

**Figure 4:**
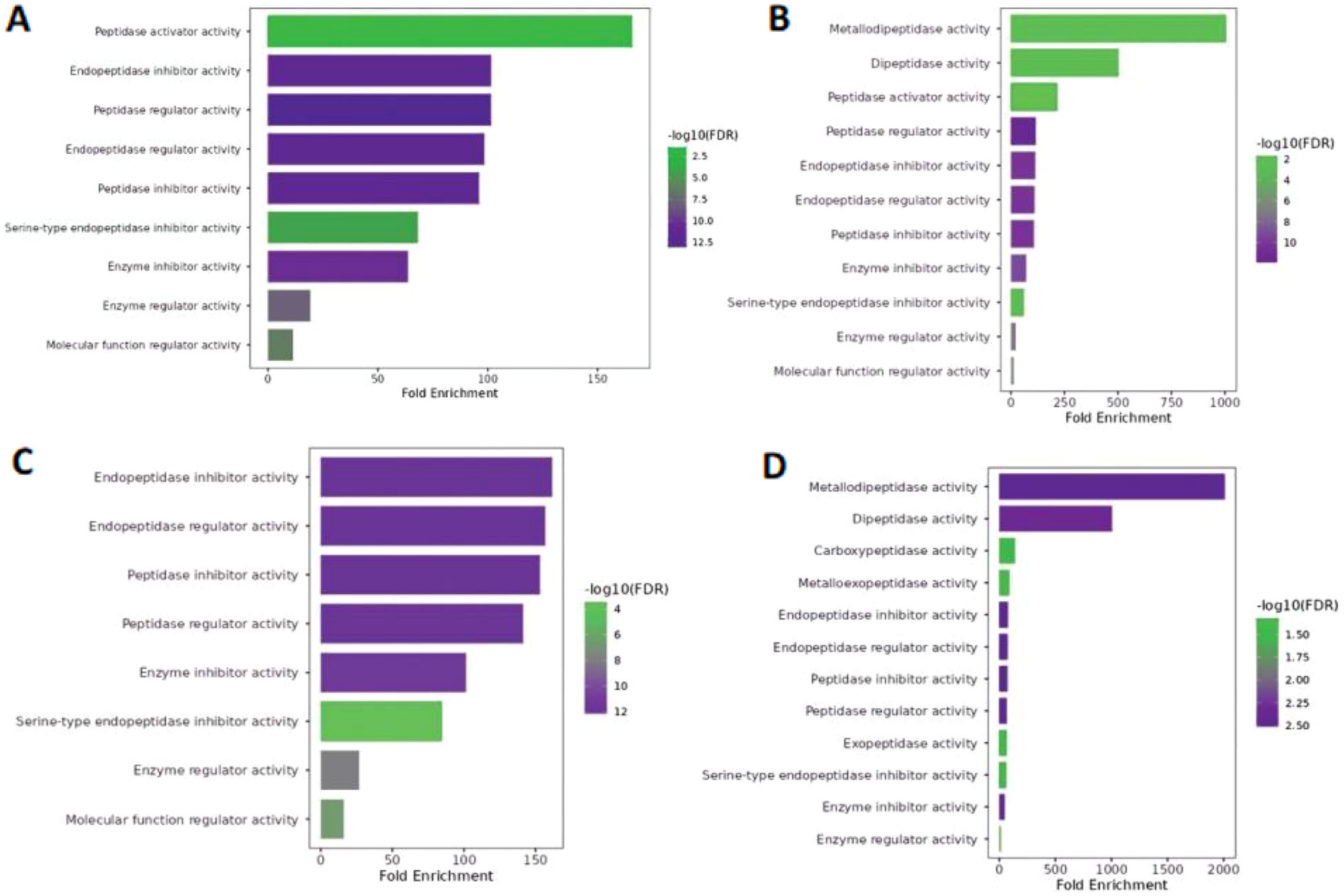
Molecular function of Hilsa samples; **(A)** denotes- sample H3 (IV-year class), **(B)** denotes sample H28 (III-year class), **(C)** denotes sample H29 (II-year class) and **(D)** denotes- sample H34 (I-year class).

In the case of molecular function, year class I (**Figure 4A**) and III hilsa showed massive variation in fold enrichment value and −log10 (FDR), with the colour reflects 2.5 and 10 being the highest, respectively, which is depicted in both graphs (**Figures 4B, D**). Notably, the main 12 and 13 GO terms related to molecular functions were enriched in class III and II hilsa, with the highest Fold Enrichment value showing metallodipeptidase activity (**Figure 4C**). Furthermore, molecular function regulatory activity was the most common lowest function expressed in all three-year classes of hilsa (year classes II, III, and IV), but in the case of year classes II and IV, peptidase activator with minimum −log10(FDR) value 4 and 2 and endopeptidase inhibitor activity, the highest −log10(FDR) value was 12 and the highest fold enrichment value, respectively. Detailed information is provided in **Supplementary File 2**.

**Figure 5:**
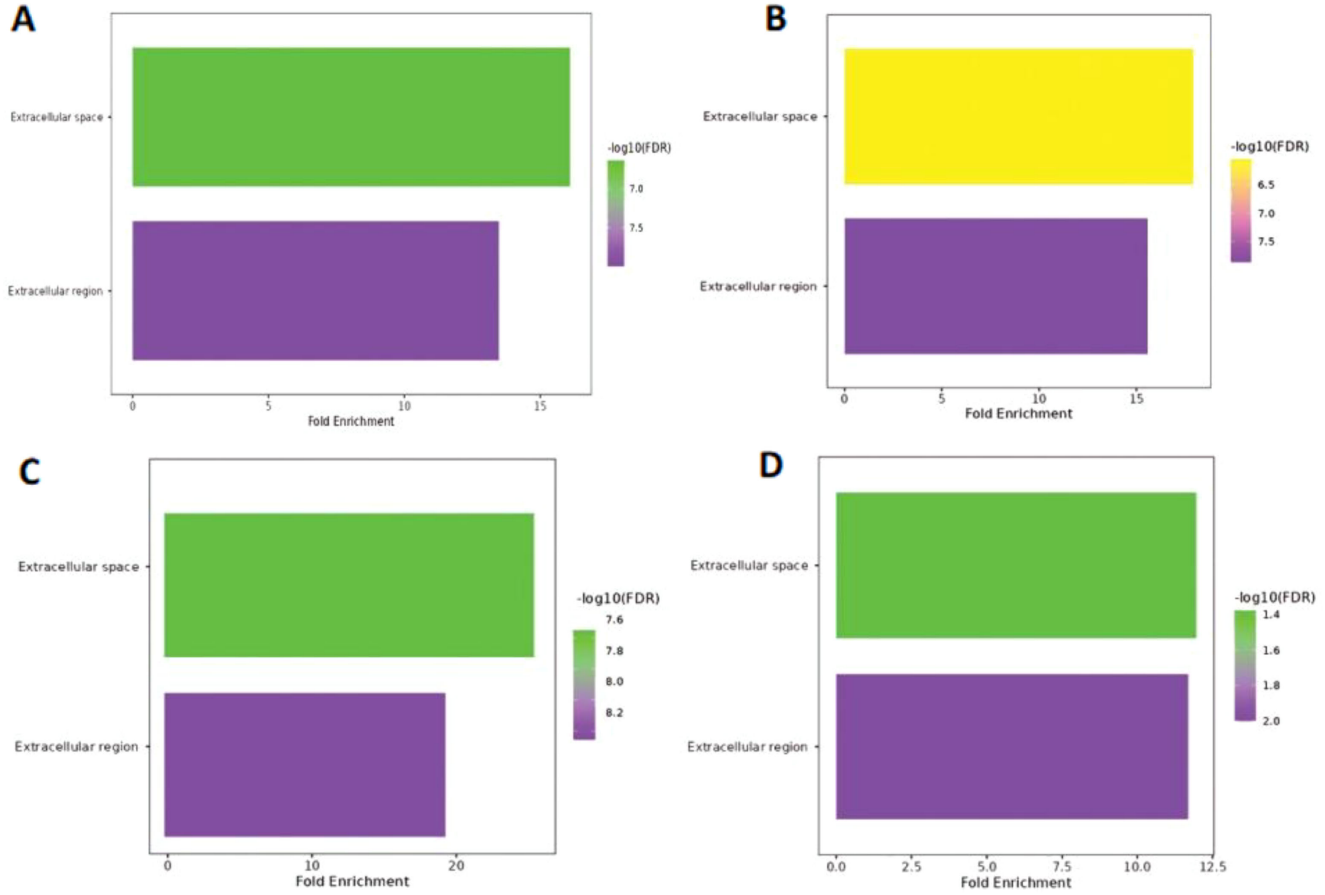
Cellular component of Hilsa samples; **(A)** denotes- sample H3 (IV-year class), **(B)** denotes sample H28 (III-year class), **(C)** denotes sample H29 (II-year class) and **(D)** denotes- sample H34 (I-year class).

Extracellular space and extracellular region were the only two GO terms identified in the cellular component; the only variable that changed was the −log10(FDR) value. A lower weight group among them exhibited low −log10(FDR) values (1.4–2.0), whereas three further samples revealed high −log10(FDR) values ranging from 6.5–8.2 (**Figure 5**). Furthermore, the extracellular space exhibited a more significant fold enrichment but a lower −log10(FDR). In contrast, the extracellular region displayed the opposite trend. Detailed information is provided in **Supplementary File 2**.

#### 3.2.1 Common and unique enriched pathways

**Figure 6:**
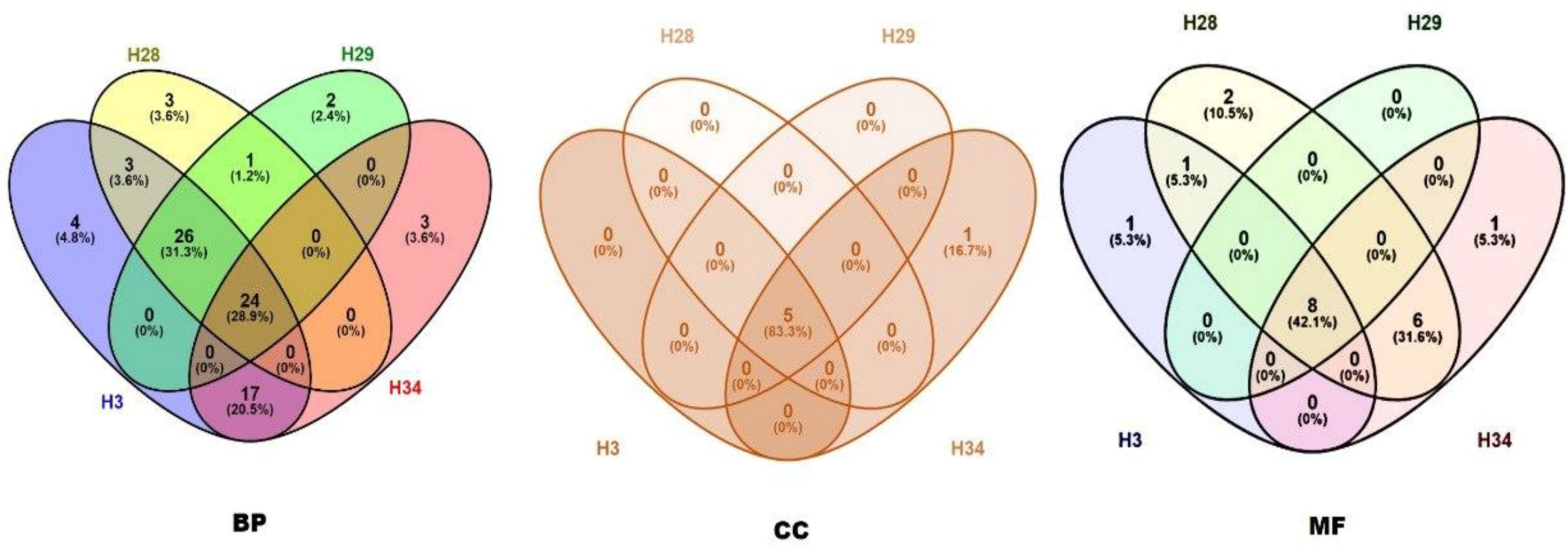
Common and unique pathways of biological process (BP), cellular component (CC) and molecular function (MF) between all samples (H3- year class I, H28- year class II, H29- year class III and H34- year class IV).

The Venn Diagram tool was preferred to detect common and unique GO terms for analysing the hilsa in each year class (**Figure 6**). In the biological process category, 24 common terms were encircled for all samples. Three samples for year classes IV, III, and II were found 26, and 17 common pathways were found between classes IV and I. There were also some unique pathways for each sample (year classes IV, III, II, and I showed unique pathways 4, 3, 2, and 3, respectively). In the case of cellular components, five common and one unique component were found in the sample year class I. Therefore, eight common pathways were identified in the molecular function of gene ontology. Detailed information is provided in **Supplementary File 2**.

### 3.3 KEGG pathway enrichment analysis

**Figure 7:**
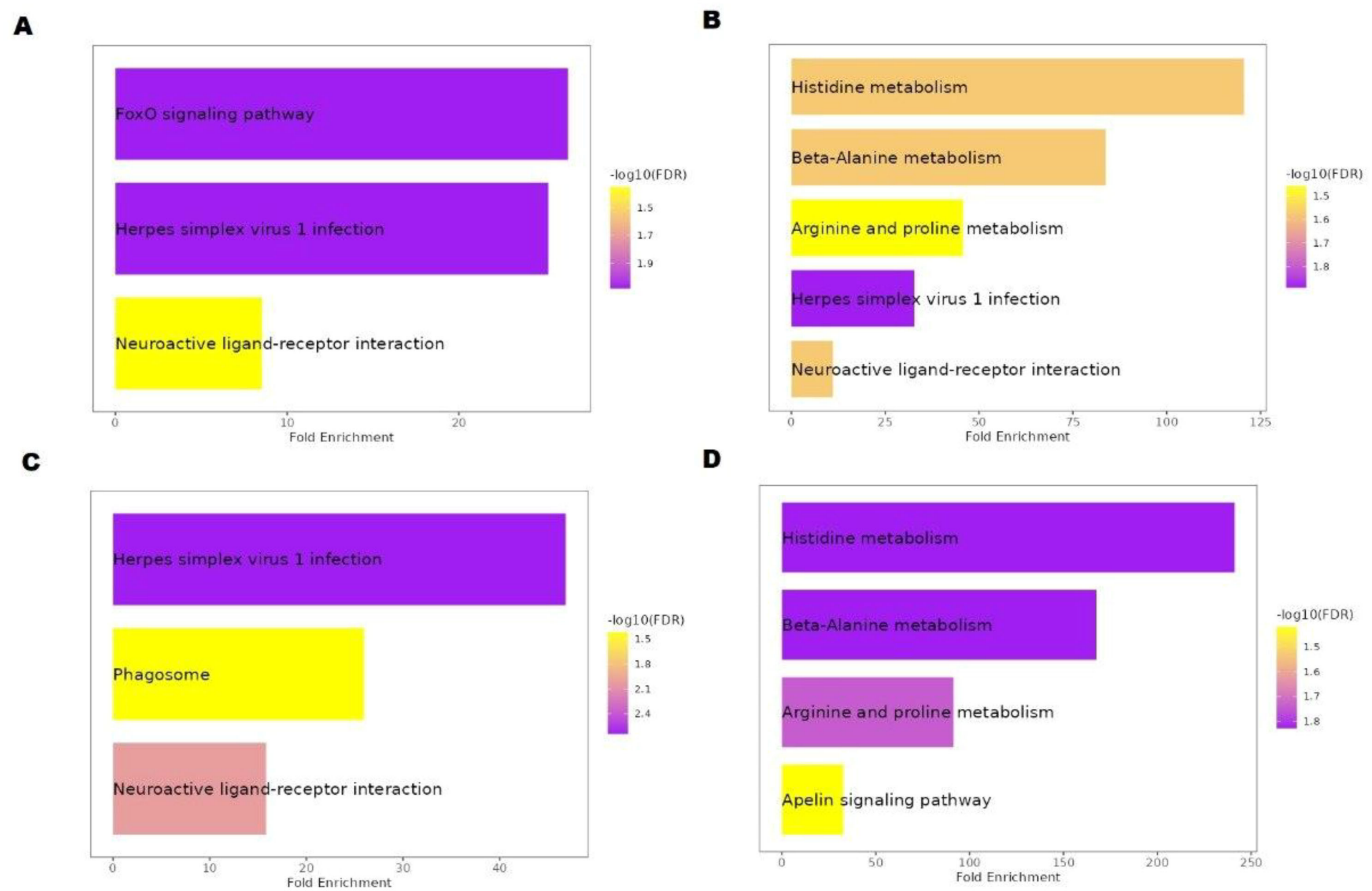
KEGG pathway analysis of Hilsa; **(A)** denotes IV-year class, **(B)** denotes III-year class, **(C)** denotes II-year class and **(D)** denotes I-year class.

In this study, pathway enrichment analysis revealed nine enriched KEGG pathways in hilsa serum samples (**Table 2**). Six genes, *sgk1, sgk3, c3a.2, c5, mef2d, and cndp2* were identified, which are involved in these pathways, providing insights into the immunology and breeding-related information of *T. ilisha* serum of different year classes. Two sample gene-enriched pathways were found in this study. The FoxO, mTOR, and apelin signalling pathway-related genes were *SGK3, sgk1*, and *mef2d* respectively (**Figure 7**).

**Table 2:** KEGG pathway mapping for *Tenualosa ilisha* serum proteome.

Sample	Gene	Enriched Pathway Name
**H3** **(year class I)**	*sgk1, sgk3, c3a.2, c5, mef2d*	• FoxO signalling pathway • Herpes simplex virus 1 infection • Neuroactive ligand-receptor interaction • Phagosome • mTOR signalling pathway • Apelin signalling pathway
**H28** **(year class II)**	*sgk3, c3a.2, c5, cndp2*	• FoxO signalling pathway • Herpes simplex virus 1 infection • Neuroactive ligand-receptor interaction • Phagosome • Arginine and proline metabolism • Histidine metabolism beta-Alanine metabolism
**H29** **(year class III)**	*c3a.2, c5*	• Herpes simplex virus 1 infection • Neuroactive ligand-receptor interaction • Phagosome
**H34** **(year class IV)**	*mef2d, cndp2*	• Histidine metabolism • beta-Alanine metabolism • Arginine and proline metabolism • Apelin signalling pathway

### 3.4 Protein association study

**Figure 8:**
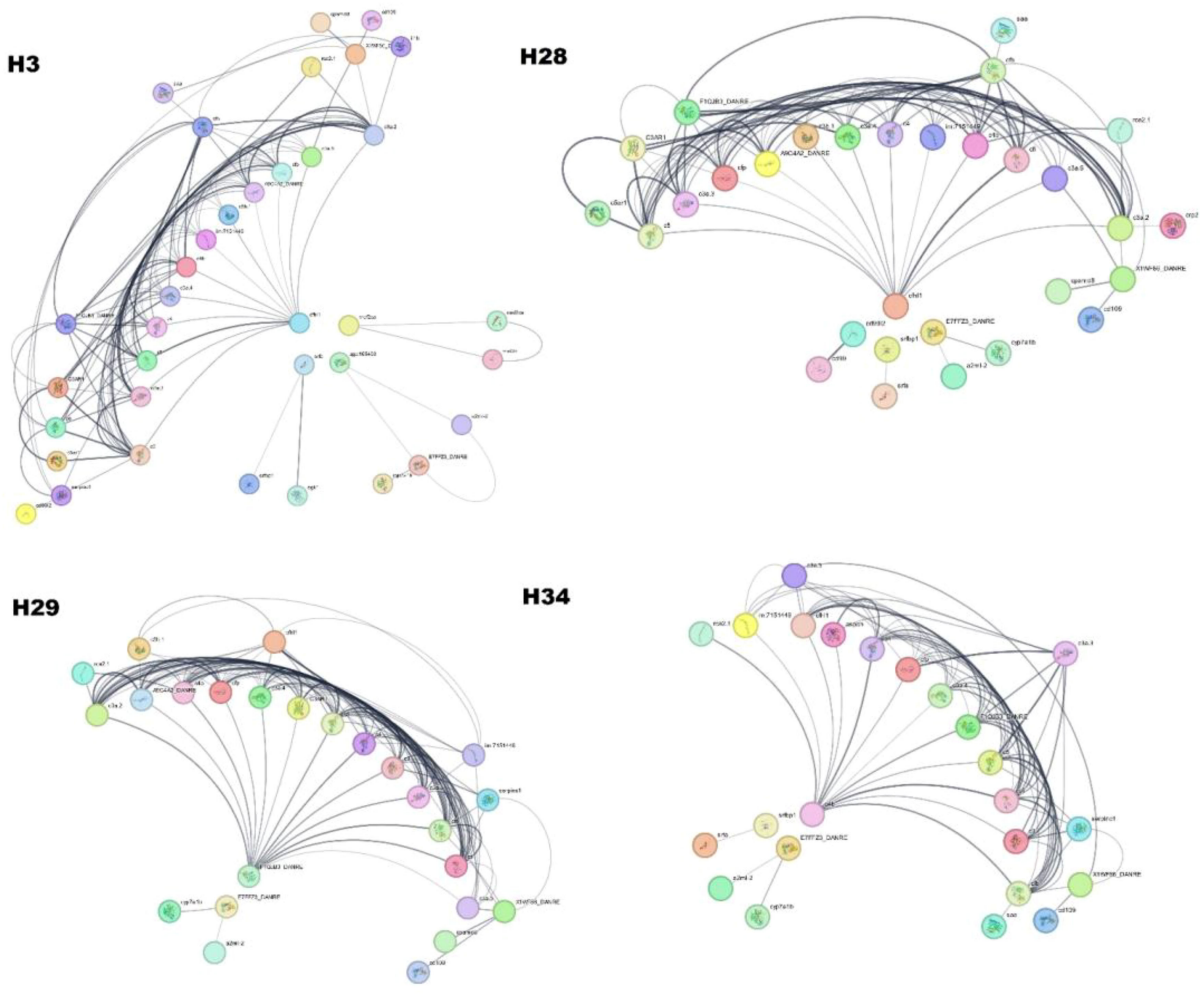
Protein-Protein Interaction of Hilsa serum proteome (H3- year class I, H28- year class II, H29- year class III and H34- year class IV).

To understand functional protein associations, PPI network analysis of the samples was performed using the STRING database (**Figure 8**). The PPIs of year class IV hilsa involved 37 directly connected nodes, with an average local clustering coefficient of 0.429 and a PPI enrichment p-value of <1.0e−16. In addition, four (one big and three small) clusters were formed; the cfi protein, which is related to the regulation of the immune system process, is associated with the maximum number of immunology-related proteins, such assrfbp1, E7FFZ3_DANRE, c3a.5, cd109, c3a.4, c4, c3a.3, c4b, sgk1, c5, and zgc:165453. Only two clusters were formed in class II with 25 directly connected nodes, with the same proteins as in class IV. Three clusters were formed in the year class I sample, the same as year class IV, but with 24 connected nodes; the same proteins were found in other samples, but one protein was found uniquely named aspdh. In the case of year Class III, 30 connected nodes are present. The cfhl1 protein was associated with the maximum protein level. In addition, one new protein and its isoform were found in the year class III of Hilsa viz. cd99 and cd9912. The remaining proteins were also found in other female hilsa samples.

## 4 Discussion

Biochemical alterations in hilsa are primarily caused by changes in environmental conditions. Overall biochemical changes are significantly influenced by various environmental and biological factors, such as age, sex, and reproductive methods. With the advancement biology in the post-genomic era, proteomic techniques have emerged as powerful tools for understanding various functions associated with signalling pathways, and many associated proteins are linked with one or more complex pathways. In this context, investigating network biology and MS-based proteomics can significantly bring a new dimension to the complex nature of biomolecules and macromolecules and their role in important nutrients (26). In fisheries, staggered proteome information, particularly on flesh quality, fish growth, behaviour, and biochemical changes is available (8–10) and mostly on zebra fish (*Danio rerio*). This study is equally important for commercially high-value species, including hilsa. Few studies have focused on the salmon ovarian fluid proteome (27), plasma proteome for immunization (28), and hilsa, and some protein studies related to flesh quality have been attributed to the muscle proteome (14). Results from the evaluation of different functions, maximum redundant protein entries, and a preliminary map of the hilsa serum interactome showed functional GO enrichment in serum investigations. This method provides a previously unattainable interatomic perspective that reveals the various biological functions of serum proteins. Based on the UniProtKB sequence database, hilsa serum contains more proteins, and their functions were first reported in this initial study of the hilsa serum proteome following advanced LC-MS/MS techniques. The total proteome of the four-year classes of hilsa reflects differential protein expression. Furthermore, 3–4% of the identified proteome comprised unnamed or uncharacterized proteins. More than 952 proteins were identified and annotated. The most frequently detected proteins were complement proteins.

In this analysis, 19 proteins with molecular functions and 75 proteins responsible for biological functions were identified. Based on biological functional annotation through GO, the year class III fish shared common GO terms, such as negative regulation of endopeptidase activity, negative regulation of peptidase activity, proteolysis, negative regulation of metabolic processes, negative regulation of catalytic activity, and regulation of hydrolase activity (**Figures 3A–C**; **Supplementary File 2**). However, class I hilsa showed four enriched GO pathways based on the FDR score, which was completely unique from other sample biological processes and reflects mostly myosin filament-related pathways, which play a role in muscle contraction and movement, including cell division, which is crucial for the early year class of life (29). Dominant peptidase and endopeptidase activities were linked to fish immunology based on GO analysis. It has broad-spectrum antimicrobial activity, and the genes that control it are highly responsive to innate immunostimulatory chemicals and microorganisms (30).

Fish are vulnerable to various biological threats, including parasites, bacteria, fungi, and viruses (31). However, owing to their unique and intrinsic properties, they can prevent microbial invasion (32, 33). In *T. ilisha* species, based on KEGG pathway analysis, six immune-related genes responsible for many pathways were identified in this study (**Table 2**). According to Gross et al. (34) and Wang et al. (35), the FoxO signalling pathway is found in higher-year classes that play important roles during migration-related stress. FoxO is essential for many distinct cellular functions, including differentiation, apoptosis, autophagy, metabolism, inflammation, and stress resistance; however, Huang et al. (36) stated that it also plays a role in heat stress. Notably, in hilsa, this pathway was enhanced at high temperatures, indicating a possible connection between altered energy metabolism and thermal adaptation during sea-to-river migration. According to the results of previous research, fish must have high readily available energy to survive at both hot and cold temperatures (37). In our investigation, the mTOR signalling pathway, which was not prominent in KEGG (**Table 2**) but present in year class IV fish, was essential for immunology. In addition, the same line of study was discovered in adult hilsa by Rommel et al. (38), and the gene responsible for this was *mef2*. Herpes simplex virus 1 infection was another enriched pathway expressed in year classes II, III, and IV which was overrepresented in earlier reports that fish, such as Tilapia, Salmon, rainbow Trout, Eel, were also affected by this virus (39). According to Wolf and Taylor (40), the herpes virus was initially identified in 1971–1975 during rainbow trout (*Oncorhynchus mykiss*) spawning time in the Washington State Winthrop fish hatchery. Additionally, the virus was identified and classified as *Herpesvirus salmonis* (41).

According to Chng et al. (42) and Pauli et al. (43), Elabela or zebrafish ELA, is expressed at a different time during embryogenesis than apelin and function as an early developmental signal that controls angioblast migration, heart development, and meso-endodermal cell movement in hilsa higher and lower year class. It might be functioning in early organ development in small hilsa and signal development and control in migration in matured hilsa. Notably, unlike in zebrafish, this pathway signalling receptor is also found in tilapia and humans (44). This work is crucial for understanding biological processes, which were derived from string analysis based on the background database of zebrafish, where 30–45% protein was mapped against hilsa peptide sequences derived by LC-MS/MS. The phagosomal pathway helps in the oxidative stress response, which is related to fish defence mechanisms and increases fish survival. This was highlighted in most year classes, except for class I. According to Kumar et al. (45), this pathway is actively involved in rainbow trout during *Yersinia ruckeri* infection. Additionally, these pathways could be helpful in comprehending biological functions and protein associations in PPI network analysis. Twenty-six proteins showed a strong connection to one another using STRING databases and applying PPI network analysis in year class I. In this connection, most proteins are complement proteins, which aid in immunology.

To the best of our knowledge, this is the first report of hilsa serum proteomics; earlier, only whole-genome sequencing of hilsa (Accession no. PRJNA422030 and genome size 146.3 Mb) were submitted to the NCBI by Mohindra et al. (46). However, further research is necessary for the systematic compilation of this proteomic analysis of hilsa serum. Furthermore, fish composition significantly fluctuated with season. This fundamental query focused on the immunological state of ethe fish throughout its migration through its energy metabolism. Fish require high energy to swim from an estuary at such a distance. Proteins and lipids are used as fuels in energy metabolism, and amino acids are ultimately affected by protein breakdown. The protein utilization and energy budget of fish during migration were determined by a detailed examination of energy metabolism and protein turnover during migration. Therefore, multiple entries for several identified proteins were detected. This result was anticipated because multiple isoforms human serum protein paralogs are present in fish plasma (47, 48). Members of this large cluster exhibited the highest frequency of immune-related proteins during freshwater migration. In this time course, the complement system proteins were annotated as c3, c4, c5, c9, and other complement proteins including their isoforms. For instance, the c3 protein and its isoforms, which are central components of the complement system, were present in significantly higher numbers in all the samples. Similarly, complements c4 and c5 were also dominant and acted in immune and inflammatory responses. Alpha macro globin found frequently work as an anti-protease to another protein named serum albumin, which is a non-specific protein carrier found in hilsa and also one of the top 10 most abundant proteins in sockeye salmon serum (49) but not detected in either zebra fish (47) or white sucker (48). In humans, albumin constitutes half of the plasma protein content (50), which means that mammalian studies typically deplete this protein (and other abundant immune proteins) prior to MS analysis to facilitate the detection of low-abundance proteins (51). A similar high-frequency protein, fibulin, was more abundant in year class IV hilsa. Several additional proteins with known immune functions were present in a big cluster, including C type like cfp, cfb, cd99 (membranous protein), their isoforms, cfhl1, and others like serpinc1, A9C4A2_DANRE, and F1QJB3_DANRE assist in maintaining the integrity of an organism by inactivating invading organisms, pathogens, and modified tissue cells. Alpha-2-macroglobulin (A2ML) is a large plasma protein within the innate immune system of vertebrates and a broad-spectrum protease-binding protein that has remained evolutionarily conserved (52). It has been discovered in the tissue of Hilsa (46) with 12 A2M isoforms through SMRT (Single-Molecule Real-Time sequencing) sequencing (46). This novel discovery of immunology-related proteins is important for understanding cell migration. Fish are vulnerable to a range of biological agents, including parasites, bacteria, fungi, and viruses. However, owing to their unique and intrinsic properties, they can prevent microbial invasion (33). In addition, our study revealed that the top 20 genes that were functional with the above-mentioned associated proteins are graphically presented in **Supplementary File 1**, which plays a vital role in metabolic processes, ATP binding, catalytic activity, molecular function, enzyme regulation, etc., other than immunology. Important genes named *sgk1, sgk3* and *C3a.2* work in cell growth, membrane transport, proliferation, and ion channels found in class IV Hilsa, which also play a role in survival and migration. Another gene, *mef2*, found in hilsa year class I and IV, has been reported in trout (*Oncorhynchus mykiss*) (53) in a year class in which new small myofibers were apparent, also helping in neuronal cell development and differentiation, and this was regulated by class II histone deacetylases. Thus, no current information on the breeding aspect of protein levels and functions involved in these vital processes in hilsa fish is available; although some proteins (A2ML, cd109) lead to ovary maturation in fish but play a vital role in pregnancy in higher vertebrates. Furthermore, proteins play important function in fish domestication to strengthen advance understanding, in our study identified several proteins and genes linked to the immune system would advance our knowledge of the immune system of hilsa self-defence mechanisms. The integrated global proteomics results and the bioinformatics analysis of the hilsa serum proteome, as shown in this study, demonstrate the viability of this approach to provide a thorough understanding of this immunological aspect to prevent naturally occurring diseases. This approach can help elucidate the relationship between individual hilsa by their weight group, either in their constitutive state or following survival challenges and conservation. Thus, knowledge of the hilsa immune system-related proteins and genes may be crucial for developing disease management plans, and further research is required.

## 5 Conclusions

This study provides the first detailed information about the different functions of age-specific protein expression related to the biological, molecular, and cellular functions of the female hilsa. Furthermore, it provides insights into the immunity-involved proteins crucial for migration and ovulation. Additionally, the discovery of genes such as *sgk1, sgk3, c3a.2, c5, mef2d* and *cndp2* linked with FoxO, mTOR, and the apelin signalling pathways provide a foundation for understanding the metabolic pathways of anadromous species. Hilsa domestication is very important considering consumer preferences and prices. Hormonal regulation and the proteins responsible for ovulation could be corroborated and manipulated, and introducing hilsa into aquaculture would be beneficial.

## Data Availability

The original contributions presented in the study are included in the article/**Supplementary Material**. Further inquiries can be directed to the corresponding author.
