# Supplementary material for: Age-specific changes in the serum proteome of female anadromous, hilsa Tenualosa ilisha: a comparative analysis across developmental stages
Source: Front Immunol. 2024 Oct 18;15:1448627. doi: 10.3389/fimmu.2024.1448627 (PMC11527666; doi:10.3389/fimmu.2024.1448627)
Supplement: Supplementary file 1 [file Table1.docx]

Table 1. Top 10 most observed proteins and their functions of female Hilsa

| **SL No.** | **Proteins** | **Functions** |
| --- | --- | --- |
|  | Alpha-2 Macroglobin | Anti Protease |
|  | Fibulin | Mesenchymal cell migration and epidermal–dermal junction formation |
|  | MEF2 | Muscle development mostly skeletal and cardiac |
|  | C4 | Immune/inflammatory response, lectin pathway |
|  | Serum Response Factor (SRF) | Cellular activities including cell growth, differentiation, cell migration and apptosis |
|  | CD109 | Regulation of gene expression, metabolic process and pregnancy related to higher vertebrate |
|  | C5 | Immune/inflammatory response later stage |
|  | Albumin | Non-specific carrier protein |
|  | non-specific serine/threonine protein kinase (sgk) | Response to cortisol and neurogenesis and GR function |
|  | C3 | Immune/inflammatory response |


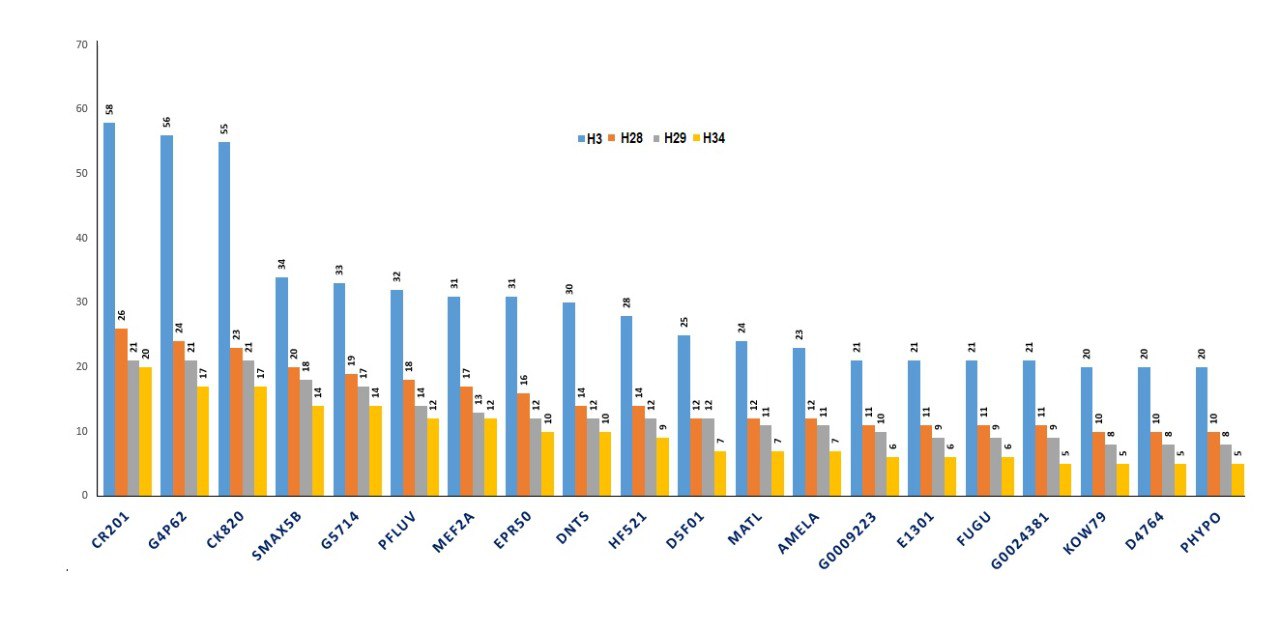


Fig 1. Graphical presentation of top 20 functional genes associated with protein
